# Yeast Extract and Silver Nitrate Induce the Expression of Phenylpropanoid Biosynthetic Genes and Induce the Accumulation of Rosmarinic Acid in *Agastache rugosa* Cell Culture

**DOI:** 10.3390/molecules21040426

**Published:** 2016-03-29

**Authors:** Woo Tae Park, Mariadhas Valan Arasu, Naif Abdullah Al-Dhabi, Sun Kyung Yeo, Jin Jeon, Jong Seok Park, Sook Young Lee, Sang Un Park

**Affiliations:** 1 Department of Crop Science, Chungnam National University, 99 Daehak-ro, Yuseong-gu, Daejeon 34134, Korea; harusarinamu@gmail.com (W.T.P.); agape0619@naver.com (S.K.Y.); jeonjin519@gmail.com (J.J.); 2 Department of Botany and Microbiology, Addiriyah Chair for Environmental Studies, College of Science, King Saud University, P. O. Box 2455, Riyadh 11451, Saudi Arabia; mvalanarasu@gmail.com (M.V.A.); naldhabi@ksu.edu.sa (N.A.A.); 3 Department of Horticulture, Chungnam National University, 99 Daehak-ro, Yuseong-gu, Daejeon 34134, Korea; jongseok@cnu.ac.kr; 4 Regional Innovation Center for Dental Science & Engineering, Chosun University, 309 Pilmun-daero, Dong-gu, Gwangju 501-759, Korea

**Keywords:** *Agastache rugose*, yeast extract, silver nitrate, phenylpropanoid biosynthetic genes, rosmarinic acid

## Abstract

The present study aimed to investigate the role of yeast extract and silver nitrate on the enhancement of phenylpropanoid pathway genes and accumulation of rosmarinic acid in *Agastache rugosa* cell cultures. The treatment of cell cultures with yeast extract (500 mg/L) and silver nitrate (30 mg/L) for varying times enhanced the expression of genes in the phenylpropanoid pathway and the production of rosmarinic acid. The results indicated that the expression of *RAS* and *HPPR* was proportional to the amount of yeast extract and silver nitrate. The transcript levels of *HPPR* under yeast extract treatment were 1.84-, 1.97-, and 2.86-fold higher than the control treatments after 3, 6, and 12 h, respectively, whereas *PAL* expression under silver nitrate treatment was 52.31-fold higher than in the non-treated controls after 24 h of elicitation. The concentration of rosmarinic acid was directly proportional to the concentration of the applied elicitors. Yeast extract supplementation documented the highest amount of rosmarinic acid at 4.98 mg/g, whereas silver nitrate addition resulted in a comparatively lower amount of rosmarinic acid at 0.65 mg/g. In conclusion, addition of yeast extract to the cell cultures enhanced the accumulation of rosmarinic acid, which was evidenced by the expression levels of the phenylpropanoid biosynthetic pathway genes in *A. rugosa*.

## 1. Introduction

Production of useful metabolites such as alkaloids, flavanoids, phenolic compounds and other phytochemicals from *in vitro* plant cell cultures has attracted many pharmaceutical industries because of advances in technology for the bulk production of important novel metabolites [1,2]. Many modern methods, including precursor feeding, elicitation, and membrane permeabilization, have been applied to enhance the level of secondary metabolites synthesized in plant cell cultures [3,4,5]. *In vitro* plant cell cultures, the use of biotic (yeast, bacteria, and fungi) and abiotic (jasmonic acid, arachidonic acid, methyl jasmonate, and other chemicals) elicitors has been reported to stimulate the production rate of useful secondary metabolites at a commercial scale in shorter production periods [6]. Elicitors derived from fungi were used to stimulate the production of secondary metabolite. This strategy is efficient in stimulating the synthesis of important secondary metabolites, such as flavonoids [7], alkaloids [8], terpenoids [9], and coumarin derivatives [10]. Rosmarinic acid produced by the phenylpropanoid pathway in medicinal plants exhibits antiviral, antimicrobial, anti-inflammatory, antioxidant, antihypertensive, and antiatherogenic activities [11,12,13,14,15,16,17,18]. 

**Figure 1:**
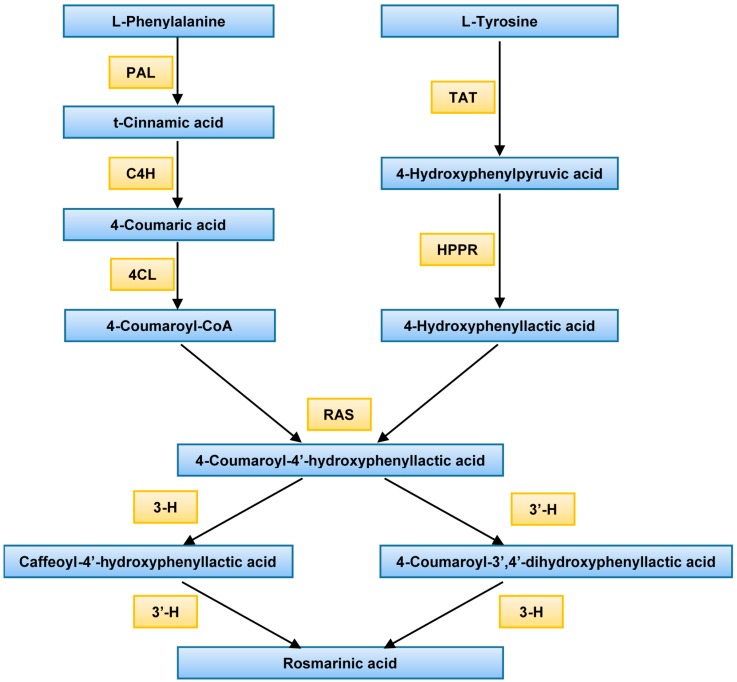
Proposed biosynthetic pathway of rosmarinic acid. PAL, phenylalanine ammonia-lyase; C4H, cinnamate 4-hydroxylase; 4CL, 4-coumarate CoA ligase; TAT, tyrosine amino transferase; HPPR, hydroxyl phenylpyruvate reductase; RAS, rosmarinic acid synthase (hydroxycinnamoyl-CoA:hydroxyphenyllactate hydroxycinnamoyl transferase); 3-H, 3-hydroxylase; 3′-H, 3′-hydroxylase.

The presumed phenylpropanoid metabolic biosynthetic pathway for the synthesis of rosmarinic acid in plants is presented in Figure 1. Phenylalanine is converted into cinnamic acid, 4-coumaric acid, and other metabolites by three enzymes: phenylalanine ammonia-lyase (PAL), cinnamate 4-hydroxylase (C4H), and 4-coumarate:CoA ligase (4CL) [19]. The activated intermediate molecule, 4-coumaroyl-CoA, serves as a precursor for the formation of a number of natural products, such as lignin, coumarin, flavonoids, and cell wall-bound phenolics. Further, 4-coumaroyl-CoA reacts with 4-hydroxyphenyllactic acid to form 4-coumaroyl-4′-hydroxyphenyllactic acid. Finally, rosmarinic acid is derived from the hydroxylation reaction between the compounds caffeoyl-4-hydroxyphenyllactic acid and 4-coumarotil-3,4-dihydroxyphenyllactic acid [20,21].

In general, higher amounts of rosmarinic acid are present in undifferentiated tissue-cultured cells than in the wild plants [22]. The first plant cell suspension cultures found to accumulate rosmarinic acid were derived from *Coleus blumei* [22,23] and rosmarinic acid accumulated up to 20% of the cell dry weight [24]. In cell suspension cultures of *Agastache rugosa* grown in B5 cultivation medium with 2 mg/L 2,4-D, and 0.1 mg/L 6-benzylaminopurine, higher cell growth and rosmarinic acid production occurred compared to 2 mg/L 2,4-D single treatment [25], whereas *Ocimum basilicum* documented 10 mg/g dry weight rosmarinic acid [26]. Rosmarinic acid content increased when the cells were treated with a benzothiadiazole elicitor preparation in *A. rugosa* [27]. Rosmarinic acid accumulation was also induced by the addition of yeast extract to suspension cultures of *Lithospermum erythrorhizon* [28] and *Orthosiphon aristatus* [29]. Rosmarinic acid accumulation as well as total phenolic content of *Salvia miltiorrhiza* hairy roots was increased by both elicitors (yeast extract and silver nitrate), but more significantly by yeast extract [30].

*Agastache rugosa*, also known as Korean mint, is a perennial herb that belongs to the mint family (Labiatae). Rosmarinic acid is considered to be the main phenylpropanoid and has been proven to contribute to the medicinal potential of *A. rugosa*. The present study was conducted to examine the effects of yeast extract and silver nitrate on the enhancement of rosmarinic acid in cell cultures of *A. rugosa*, as well as to determine whether the addition of yeast extract and silver nitrate influence the phenylpropanoid biosynthetic pathway genes in *A. rugosa*.

## 2. Results

### 2.1. Gene Expression

**Figure 2:**
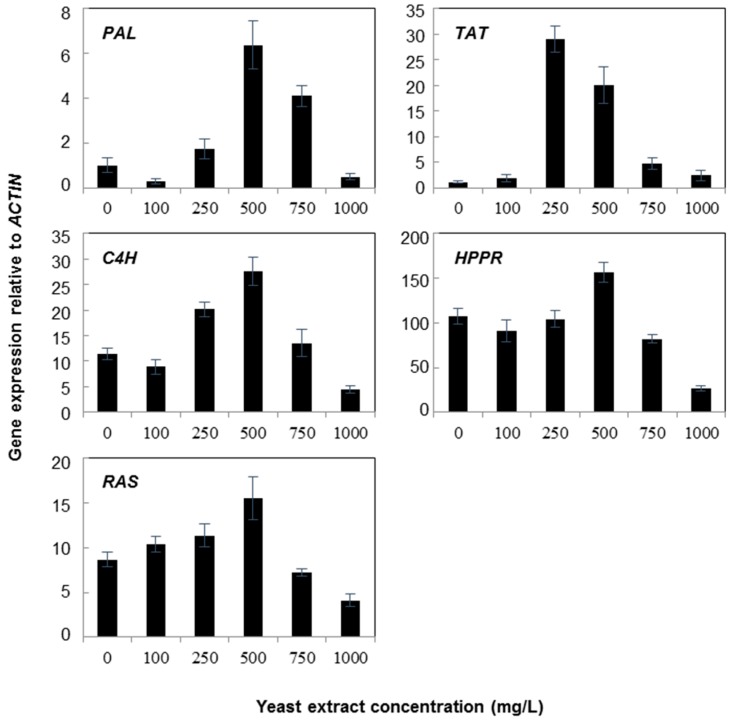
Expression of rosmarinic acid biosynthetic genes in *Agastache rugosa* calli treated with various concentrations of yeast extract for 72 h. Transcription levels for each gene were analyzed relative to that of *ACTIN*. Error bars show standard deviation values.

**Figure 3:**
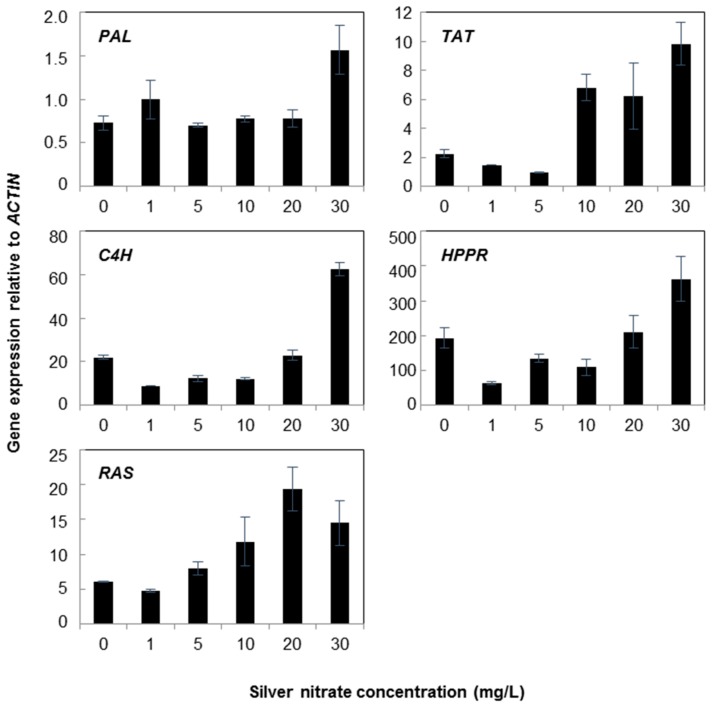
Expression of rosmarinic acid biosynthetic genes in *Agastache rugosa* calli treated with various concentrations of silver nitrate for 72 h. Transcription levels for each gene were analyzed relative to that of *ACTIN*. Error bars show standard deviation values.

Ten-day-old cell suspension cultures of *A. rugosa* were treated with various concentrations of yeast extract or silver nitrate and the changes in the expression levels of rosmarinic acid biosynthetic genes were investigated. The expression levels of phenypropanoid genes under various concentrations of yeast extract and silver nitrate are presented in Figure 2 and Figure 3. The yeast extract treatments influenced greatly the expression of different genes in the cell suspensions of *A. rugose* (Figure 2). In particular, 500 mg/L yeast extract induced higher levels of mRNA for all genes compared to other concentrations. *RAS* transcription levels were directly proportional to the concentration of yeast extract up to 500 mg/L. The highest and lowest levels of *RAS* transcript were obtained in cell suspensions treated with 500 mg/L and 1000 mg/L yeast extract, respectively. The results of *HPPR* expression were similar to that of the *RAS* gene. Although the highest *HPPR* transcript level was in suspension cells treated with 500 mg/L yeast extract, the increase was not significant. The results indicated that the expression level of *C4H*, *PAL* and *TAT* were maximum at 500 mg/L yeast extract, whereas the lowest transcript levels were found in cell suspensions treated with 1000 mg/L yeast extract.

In another experiment, silver nitrate significantly affected the expression levels of genes in cell suspensions of *A. rugosa* at concentrations less than one-tenth that of yeast extract (Figure 3). The transcript levels of most of the genes increased upon silver nitrate treatments. In particular, cell suspensions treated with 30 mg/L silver nitrate showed the highest levels of mRNA for all tested genes, but the expression of *RAS* decreased slightly at a concentration of 30 mg/L silver nitrate. At 30 mg/L, the transcript levels of *HPPR* and *TAT* were 1.87- and 3.05-fold higher than the non-treated control. Furthermore, *TAT* expression increased sharply in cell suspensions treated with 10, 20, and 30 mg/L silver nitrate up to levels 2.75- to 4.35-fold higher than that in the control. There was no significant change in the transcript levels of *PAL* in suspension cells treated with 20 mg/L silver nitrate, whereas the highest levels of *PAL* transcript were observed in cell suspensions treated with 30 mg/L silver nitrate, reaching levels 3.54-fold higher than those in the control.

**Figure 4:**
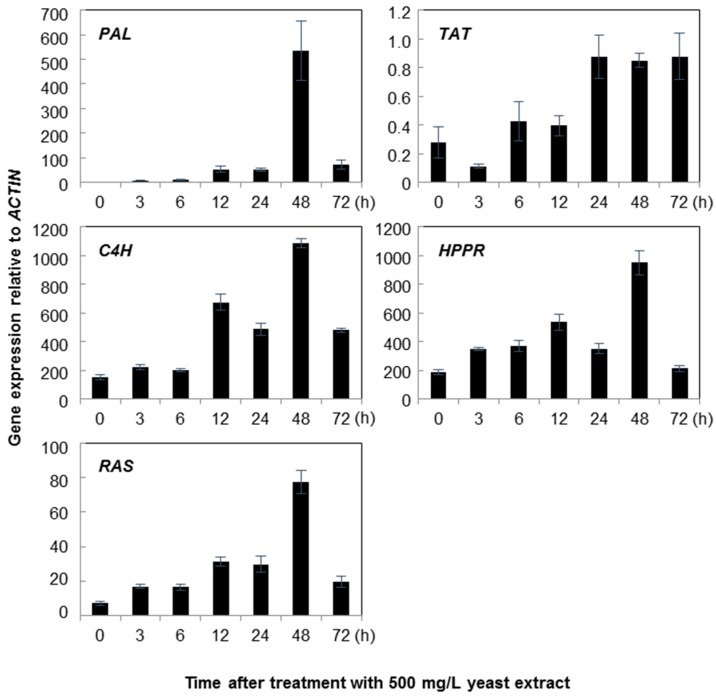
Time course of rosmarinic acid biosynthetic genes expression in *Agastache rugosa* calli treated with 500 mg/L yeast extract. Transcription levels for each gene were analyzed relative to that of *ACTIN*. Error bars show standard deviation values.

The optimum concentrations of yeast extract and silver nitrate, 500 mg/L and 30 mg/L respectively, were used to treat ten-day-old cell suspensions of *A. rugosa*, and their influence on rosmarinic acid biosynthetic genes was investigated over a period of time from 0 to 72 h. The results documented that *RAS* expression levels increased from 3 h after the yeast extract treatment until 48 h, followed by a dramatic decrease 72 h after the yeast extract treatment (Figure 4). The highest level of *RAS* expression was observed at 48 h and showed levels 10.95 times higher than that in the control treatment. However, the expression levels of *HPPR* were 1.84-, 1.97-, and 2.86-fold higher at 3, 6, and 12 h, respectively, and the expression was slightly lower after 24 h; however, the expression was 5-fold higher than the mean expression level in non-treated cell suspensions. *C4H* expression was similar to that observed for *RAS*. The expression of *TAT* and *PAL* did not significantly change until 12 and 24 h after the treatment of cell suspensions with yeast extract. The highest *TAT* and *PAL* expression in cell suspension cultures were observed after 72 and 24 h, respectively.

**Figure 5:**
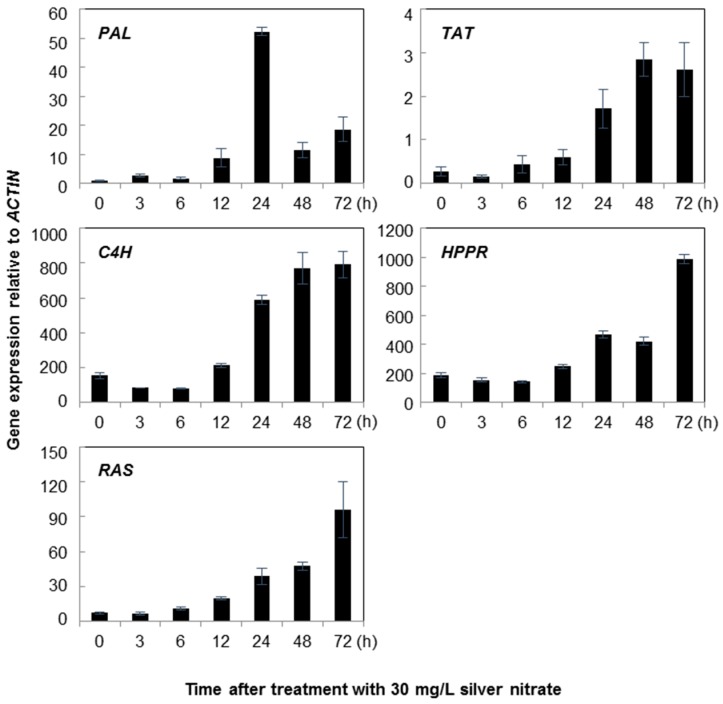
Time course of rosmarinic acid biosynthetic genes expression in *Agastache rugosa* calli treated with 30 mg/L silver nitrate. Transcription levels for each of the genes were analyzed relative to that of *ACTIN*. Error bars show standard deviation values.

Treatments with silver nitrate also influenced significantly the expression levels of rosmarinic acid biosynthetic genes in cell suspensions of *A. rugosa*. *RAS* expression levels in cell suspension culture continuously increased from 3 to 72 h after the silver nitrate treatment, with levels 13.56-fold higher than those in the control treatment (Figure 5). *HPPR* expression exhibited a similar trend as that of *RAS* gene. There was no considerable change in *C4H* expression in the first 12 h after the treatment, showing only 1.37-fold higher transcript levels compared to those in the control group. The highest amount of *C4H* transcripts in cell suspension culture was observed 72 h after the silver nitrate treatment. *TAT* expression showed a similar trend to that observed for the *C4H* gene. *TAT* and *PAL* expression levels were slightly increased in the first 12 h after the silver nitrate treatment. However, these levels sharply increased after 24 h of treatment, and at 48 h after the treatment, the expression levels were 2.86-fold higher than those in the control and remained almost constant after 72 h. The highest level of *PAL* expression was observed after 24 h and exhibited expression levels 52.31-fold higher than those in the non-treated control.

### 2.2. Quantification of Rosmarinic Acid

**Figure 6:**
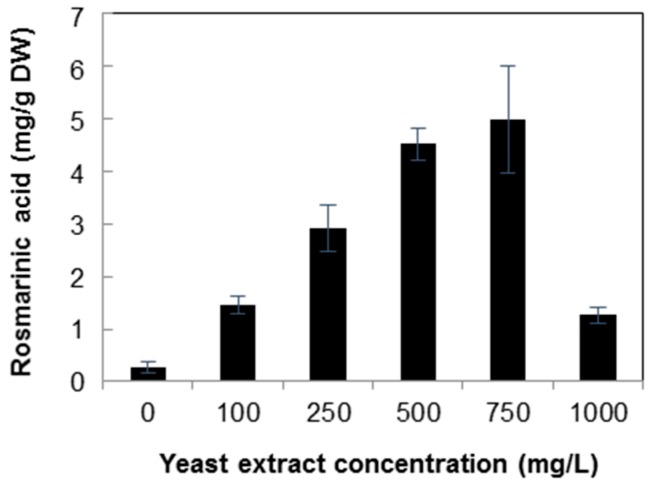
Content of rosmarinic acid in *Agastache rugosa* callus treated with various concentrations of yeast extract for 72 h. The levels of rosmarinic acid from each sample were analyzed by high performance liquid chromatography (HPLC). Error bars show standard deviation values. DW, dry weight.

Although there were differences in the contents, both yeast extract and silver nitrate treatments increased the rosmarinic acid contents at appropriate elicitor concentrations in cell suspension culture of *A. rugosa*. The amount of rosmarinic acid increased with increasing concentration of yeast extract up to 750 mg/L, and then, it dramatically decreased in the callus suspension culture treated with 1000 mg/L yeast extract (Figure 6). The rosmarinic acid contents were 1.46, 2.92, and 4.53 mg/g in cell suspensions treated with 100, 250, and 500 mg/L yeast extract, respectively. The highest rosmarinic acid content of 4.98 mg/g was observed in cell suspensions treated with 750 mg/L yeast extract, which was 18.5-fold higher than the levels found in the control and 1.10-fold higher than the levels (4.53 mg/g) found in cell suspensions treated with 500 mg/L yeast extract.

**Figure 7:**
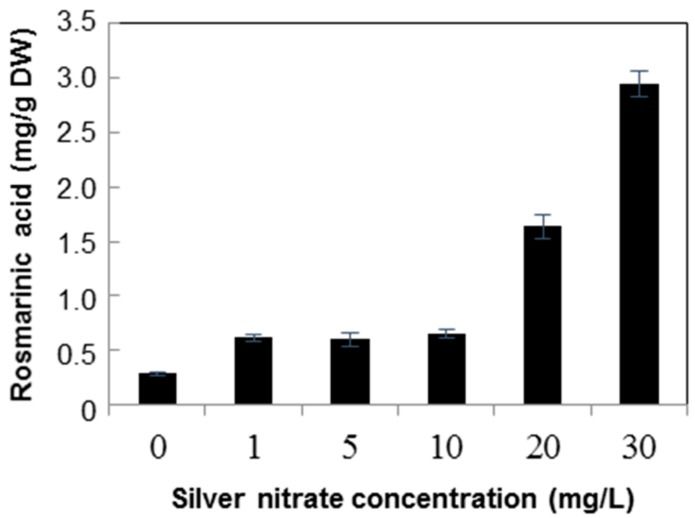
Contents of rosmarinic acid in *Agastache rugosa* callus treated with various concentrations of silver nitrate for 72 h. The levels of rosmarinic acid in each sample were analyzed by HPLC. Error bars show standard deviation values. DW, dry weight.

Silver nitrate (10 mg/L) treatment did not show a significant change in the rosmarinic acid content (0.65 mg/g) (Figure 7). However, at 20 and 30 mg/L silver nitrate, rosmarinic acid content increased to 1.64 and 2.94 mg/g, respectively, which was 10.12-fold higher than the mean content in the non-treated control. Rosmarinic acid accumulation was stimulated by increasing the concentration of yeast extract treatments to 750 mg/L, and then it noticeably decreased with 1000 mg/L, whereas in the case of silver nitrate, 10 mg/L and 20 mg/L showed better production. Nevertheless, the highest detected amount of rosmarinic acid was 4.98 mg/g in cell suspensions treated with 750 mg/L yeast extract, which was 1.69-fold higher than 2.94 mg/g of rosmarinic acid found in cell suspensions treated with 30 mg/L silver nitrate. Detailed analysis confirmed treatment with both elicitors, *i.e.*, 500 mg/L yeast extract and 30 mg/L silver nitrate, increased the rosmarinic acid concentration over time.

**Figure 8:**
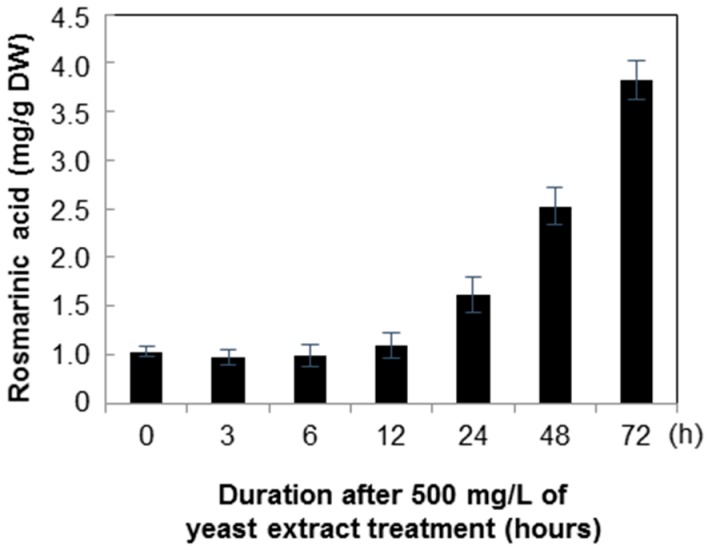
Time course of rosmarinic acid content in *Agastache rugosa* callus treated with 500 mg/L yeast extract. The levels of rosmarinic acid in each sample were analyzed by HPLC. Error bars show standard deviation values.

**Figure 9:**
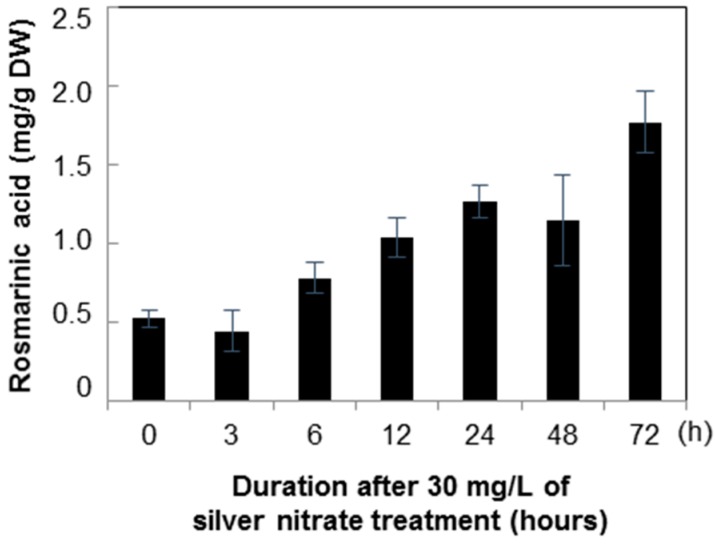
Time course of rosmarinic acid contents in *Agastache rugosa* callus treated with 30 mg/L silver nitrate. The levels of rosmarinic acid in each sample were analyzed by HPLC. Error bars show standard deviation values.

Rosmarinic acid accumulation at 24, 48, and 72 h after yeast extract treatment was 1.12, 20.02, and 3.33 mg/g respectively. After 72 h, the content was 6.30-fold higher than the mean level in the non-treated control (Figure 8). However, in the silver nitrate treatment after 6, 12, and 24 h cultivation, the concentrations of rosmarinic acid were 0.78, 1.01, and 1.12 mg/g, respectively (Figure 9). Among the treatments, the change in rosmarinic acid biosynthesis occurred faster in cell suspensions treated with silver nitrate than in those treated with yeast extract.

## 3. Discussion

The present study confirmed that the synthesis of rosmarinic acid in *A. rugosa* callus suspension cultures were stimulated by a biotic (yeast extract) and abiotic (silver nitrate) elicitor at different concentration levels. Previously, Xu *et al.* (2008, [25]) reported that higher amount of rosmarinic acid (11.5 mg/g) and maximum growth of callus tissue (7.7 g/L) were obtained in *A. rugosa*, added with 2 mg/L 2,4-D and 0.1 mg/L 6-Benzylaminopurine. Kintzios *et al.* (2003, [26])) claimed that the leaf-derived cell suspension cultures of *Ocimum basilicum* accumulated rosmarinic acid up to 10 mg/g dry weight.

The results presented here corroborate previous studies on the effect of various elicitors on rosmarinic acid accumulation in different plant species. *Coleus blumei* supplemented with *Pythium aphanidermatum* enhanced the production of rosmarinic acid [24]. Kim *et al.* (2001) [27] demonstrated that rosmarinic acid content increased yeast elicitor, and observed rosmarinic acid mainly in the cells. Yeast extract also enhanced rosmarinic acid accumulation in *Lithospermum erythrorhizon* [31] and *Orthosiphon aristatus* [29]. 

Rosmarinic acid and phenolic compound production was stimulated by both elicitors (yeast extract and silver nitrate), but more significantly by yeast extract [30], and the same results were observed in the present study on *A. rugosa* cell suspension cultures. Our results confirmed that rosmarinic acid content was enhanced up to 18.5-fold by the addition of 750 mg/L yeast extract to *A. rugosa* suspension cells compared to the levels found in non-elicited cell suspensions, and up to 10.12-fold by the treatment with 30 mg/L silver nitrate compared to that of the non-treated control. These amounts of rosmarinic acid were much higher than the previously reported levels of only 5.7-fold of those found in non-elicited cell suspensions [27].

Kim *et al.* (2001) [27] reported that the content of rosmarinic acid increased with cell growth during the stationary phase and then decreased gradually because of the browning process. In contrast, in lavandin cell suspensions, the amount of rosmarinic acid gradually decreased because of the stress caused by peroxidase-like enzymes [32]. However, the similar phenomenon was not observed in the *A. rugosa* cell suspensions in the present study because the experiments were conducted for a relatively short period. There are several biotechnological applications such as callus [1], suspension cell [25], *in vitro* shoot [33], and hairy root culture [34] used for the production of rosmarinic acid. Among these systems hairy root culture was more predominant in medicinal plants. Few elicitors hyperactively produce rosmarinic acid in *in vitro* cultures of plants [35]. 

Gene expression studies have shown a similar pattern as that of rosmarinic acid accumulation in *A. rugosa* cell suspension cultures. The expression levels of *PAL*, *C4H* and *4CL* in the cell suspensions of *A. rugosa* are involved in the secondary metabolites synthesis [36]. Simmilar results were observed in the previous studies [24,28,29,31]. Moreover, elicitor-induced biosynthesis of rosmarinic acid and phenolic compounds in *S. miltiorrhiza* hairy roots correlated with TAT activity [30]. PAL and HPPR activities increased rapidly and transiently, whereas TAT activity exhibited only a slight increase. Mizukami *et al.* (1993) observed a similar phenomenon in the results [31]. Thus, yeast extract and silver nitrate may possess the ability to promote the rosmarinic acid biosynthetic pathway, thereby increase in rosmarinic acid production in cell suspensions. However, our results were not consistent with the results of Yan *et al.* (2006), which showed that rosmarinic acid and other phenolic compounds were enhanced in *S. miltiorrhiza* hairy root cultures using different concentrations of yeast extract and silver ions [30]. Rosmarinic acid amounts in *Orthosiphon aristatus*, *Coleus blumei*, and *Lithospermum erythrorhizon* were also activated using yeast extract and methyl jasmonate as elicitors [24,28,29,31].

## 4. Materials and Methods

### 4.1. Seed Germination and Callus Induction

The seeds of *A. rugosa* were procured from Aram seed company (Seoul, Korea). For germination studies, the seeds were surface-sterilized with 70% (*v*/*v*) ethanol for 1 min and 4% (*v*/*v*) sodium hypochlorite solution with a few drops of 100% (*v*/*v*) Tween 10 min, then rinsed three times in sterilized distilled water. Seven sterilized seeds were placed on 25 mL of half MS basal solid medium in petri dishes (90 × 15 mm). The basal medium was supplemented with salts and vitamins of MS, 3% (*w*/*v*) sucrose and solidified with 0.75% (*w*/*v*) phyto agar (Duchefa, Haarlem, The Netherlands). The medium was adjusted to pH 5.8 before adding agar, and then sterilized by autoclaving at 121 °C for 20 min. Then, the seeds were germinated in a growth chamber at 25 °C under standard cool white fluorescent tubes (380 nm, SL5-SW501T-1; Sammi Elecronic Co. Ltd., Seoul, Korea) for a 16 h photoperiod.

The leaves obtained from 2-week-old plantlets after *in vitro* germination were used as explants to establish callus cultures. Leaf explants were cut aseptically at the ends into sections of approximately 7 × 7 mm^2^ in size, which were then cultured in petri dishes (90 × 15 mm) on B5 medium supplemented with 2.0 mg/L 2,4-dichlorophenoxyacetic acid (2,4-D), 0.1 mg/L kinetin, 3% sucrose, and 0.75% plant agar. Sub-cultures of callus (1.5–2.0 g) were carried out every 2 weeks.

### 4.2. Suspension Culture and Elicitor

Cell suspensions of *A. rugosa* were grown in a 100-mL Erlenmeyer flask containing 30 mL of half strength MS liquid medium with 3% (*w*/*v*) sucrose, and sub-cultured every two weeks. Then the cells were transferred into a 100-mL flask containing 30 mL medium with 10-mL cultured cells under the optimal culture conditions. For elicitor treatment, two different kinds of elicitors, *i.e.*, yeast extract and silver nitrate, were used. Each elicitor was dissolved in distilled water. Yeast extract solutions were prepared freshly and were added to the culture medium immediately at concentrations of 0, 100, 250, 500, 750, and 1000 mg/L for 72 h; silver nitrate solutions were immediately added to the culture medium at concentrations of 0, 1, 5, 10, 20, and 30 mg/L for 72 h. After three days, cell suspensions grown under each elicitor treatment were harvested and frozen in liquid nitrogen and stored at −80 °C until further use. To examine the effects of time, cell suspensions were treated under the same conditions as above for 0, 3, 6, 12, 24, 48, and 72 h in 500 mg/L of yeast extract or 30 mg/L of silver nitrate. The suspension cultures were maintained at 25 °C in a shaking incubator (HB-201SF, Han Baek Scientific Co, Bucheon, Korea) at 120 rpm. Each elicitor treatment consisted of three flasks and the experiments were repeated three times.

### 4.3. Total RNA Extraction and cDNA Preparation

Total RNA was isolated from the frozen samples using a modified Trizol method. Briefly, the harvested plantlet samples were finely ground using a mortar and pestle with liquid nitrogen. One hundred milligrams of the ground sample was dissolved in 1 mL of TRI reagent^®^ (Molecular Research Center, Inc., Cincinnati, OH, USA) together with 200 μL of chloroform for phase separation. The upper aqueous phase was gently collected and centrifuged at 13,000 rpm using a micro high speed centrifuge (Micro 17TR, Hanil Science Medical, Incheon, Korea) for 15 min at 4 °C to pellet RNA. After centrifugation, the supernatant was discarded, and the pellet was washed with 70% ethanol and re-suspended in DEPC-treated water. Finally, the integrity of RNA was determined using a NanoVue™ Plus Spectrophotometer (GE Healthcare, Buckinghamshire, UK) and formaldehyde RNA agarose gel electrophoresis.

### 4.4. Quantitative Real Time-PCR for Gene Expression Analysis

The first strand of cDNA was synthesized using 1 μg of total RNA according to the manufacturer’s instructions (ReverTraAce, Toyobo, Japan). The reverse transcribed cDNA products were used as templates for gene expression analysis with gene-specific primers detailed in Table 1. The level of expression of each gene was presented as relative expression, which is the Ct value of each gene compared to that of a housekeeping gene. qRT-PCR was performed on a CFX96 real time system (BIO-RAD Laboratories, Hercules, CA, USA) with the 2X Real-Time PCR Smart mix (BioFACT, Daejeon, Korea) by following these conditions: 95 °C for 15 min, followed by 40 cycles of 95 °C for 15 s, annealing for 15 s at 55 °C, and elongation for 20 s at 72 °C. Transcript levels were normalized relative to actin as a housekeeping gene. Three replicates of each sample were used for real-time PCR analysis and the significant differences between treatments were evaluated by standard deviation.

### 4.5. Analysis of Phenylpropanoids by HPLC

For high performance liquid chromatography (HPLC) analysis, samples were freeze-dried under vacuum for at least 48 h, ground into a fine powder using a mortar and pestle; then, each 100 mg of sample was extracted with 5 mL of 100% methanol for 1 h at 60 °C using ultrasonic waves. The phenylpropanoids, namely rosmarinic acid was extracted by methanol. After centrifugation, the supernatant was filtered through 0.45 μm PVDF filter (Whatman, GE Healthcare) and the extracts were analyzed using a HPLC system (NS-4000, Futecs, Daejeon, Korea). The analysis was monitored using a UV detector at 340 nm and performed using a reverse phase (C18, 250 mm × 4.6 mm, 5 μm) column (Prontosil, Bischoff, Germany) at 30 °C. The mobile phase was a gradient mixture of absolute methanol and water added 0.1% (*v*/*v*) acetic acid. The flow rate was maintained at 1.0 mL/min and the injection volume of each sample was 20 μL. The concentration of phenylpropanoids in the samples was calculated using a standard curve. Standard compounds were purchased from Sigma-Aldrich Corporation (St. Louis, MO, USA). Mean values were obtained from three independent replicates.

### 4.6. Statistical Analysis

For qRT-PCR and HPLC statistical analysis, data were analyzed by the statistical analysis software (SAS version 9.3, SAS Institute Inc., Cary, NC, USA). All data are given as the average mean and standard deviation of triplicate experiments. The experimental data were subjected to an analysis of variance (ANOVA), and significant differences among the means were determined using Duncan’s multiple-range test.

## 5. Conclusions

In conclusion, the present study has demonstrated that the supplementation of different concentrations of yeast extract and silver nitrate elicit and stimulate the accumulation of rosmarinic acid in *A. rugosa*, while the elicitors enhance the expression of genes involved in the synthesis of rosmarinic acid in the phenylpropanoid pathways. The expression levels of *RAS*, *HPPR* *C4H*, *PAL*, and *TAT* genes confirmed that 500 mg/L and 20 mg/L concentrations of yeast extract and silver nitrate, respectively, were optimum for the synthesis of higher amounts of rosmarinic acid. This study provides the basis for further research on improving the content of rosmarinic acid in *A. rugosa* using elicitors and hairy root induction.

## Figures and Tables

**Table 1:** Primers used to qRT-PCR analysis.

Primers	Sequences (5′ to 3′)	Amplicon Size (bp)
*ArActin F*	ACCTCAAAATAGCATGGGGAAGT	151
*ArActin R*	GGCCGTTCTCTCACTTTATGCTA	
*ArPAL F*	ACGGCTCCAACGGTCATAATAAT	108
*ArPAL R*	ATCCGCTTTACCTCCTCAAGGT	
*ArC4H F*	GTTCGAGAGTGAGAATGATCCGT	157
*ArC4H R*	ATAATCCTTGAACAATTGCAGCC	
*ArHPPR F*	AAGGGGATTAGGGTTACCAACACG	200
*ArHPPR R*	ATTCTGCCCAATCCTATGATGCC	
*ArTAT F*	AGGCAGCAGTACCAGCCATTCTT	163
*ArTAT R*	TTGACCATGAAAGCCATTGATCC	
*ArRAS F*	GGCGAACTACCACACGCTGAG	161
*ArRAS R*	CGATCTCGAGACGGTTATTGTCG	

## Abbreviations

The following abbreviations are used in this manuscript:

PAL	Phenylalanine ammonia-lyase
C4H	Cinnamate 4-hydroxylase
4CL	4-Coumarate CoA ligase
TAT	Tyrosine amino transferase
HPPR	Hydroxyl phenylpyruvate reductase
RAS	Rosmarinic acid synthase (hydroxycinnamoyl-CoA:hydroxyphenyllactate hydroxycinnamoyl transferase)
3-H	3-Hydroxylase
3′-H	3′-Hydroxylase
